# Electromodulated reflectance study of self-assembled Ge/Si quantum dots

**DOI:** 10.1186/1556-276X-6-208

**Published:** 2011-03-09

**Authors:** Andrew Yakimov, Aleksandr Nikiforov, Aleksei Bloshkin, Anatolii Dvurechenskii

**Affiliations:** 1 Institute of Semiconductor Physics, Siberian Branch of the Russian Academy of Sciences, Novosibirsk, Russia

## Abstract

We perform an electroreflectance spectroscopy of Ge/Si self-assembled quantum dots in the near-infrared and in the mid-infrared spectral range. Up to three optical transitions are observed. The low-energy resonance is proposed to correspond to a band-to-continuum hole transition in the Ge valence band. The other two modulation signals are attributed to the spatially direct transitions between the electrons confined in the *L *and Δ(4) valleys of the Ge conduction band, and the localized hole states at the Γ point.

## Introduction

In order to realize Si-based optoelectronics, Ge quantum dots (QDs) in Si matrices have attracted a large interest during the past years. Detailed knowledge on the electronic band structure and related optical transitions is very important when using self-assembled Ge/Si QDs in Si-based photonic devices. To date, most work on the optical properties of Ge/Si QDs is based on the photoluminescence (PL) spectroscopy [1-4]. However, as a rule, PL measurements provide information on the ground-state transitions only. To study high-energy excited states, it is more useful to perform absorption [5] or reflectance [6,7] experiments. In this study, the optical transitions in layers of Ge/Si QDs are investigated by electroreflectance (ER) spectroscopy as a function of applied electric field.

## Experimental details

**Figure 1:**
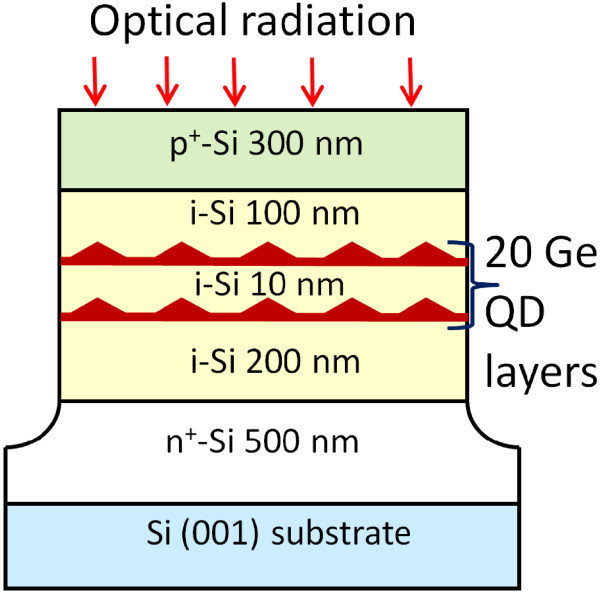
**Schematic cross section of the QD device used to make photocur-rent and ER measurements**. The structure is that of a *p*-*i*-*n *diode with 20 layers of Ge QDs in the depleted intrinsic region.

For controlled tuning of the electric field, the Ge QDs are embedded in the intrinsic region of a Si pin diode, allowing fields to be applied parallel the growth direction. To rule out spurious effects and to correctly assign the spectral features due to the presence of the dots, three sets of samples were grown by means of molecular beam epitaxy (MBE) on a *p*-Si(001) substrate with a resistivity of 150 Ω cm. The first one contains no Ge and hence can be considered as a reference sample. The second sample contains twenty Ge wetting layers (WLs), each 4 monolayer (ML) thick. A WL represents thin Ge planar layer which forms during the early stage of Ge deposition. And finally, there is a sample with 20 layers of Ge QDs lying on WLs (Figure 1). The growth temperature was generally 500°C for all layers. First, a 500-nm Sb-doped *n*^+^-type Si buffer layer with doping concentration of 5 × 10^18 ^cm^-3 ^followed by a 200-nm Si undoped layer were grown. Then 20 Ge layers separated by 10-nm Si spacer layer, followed by a 100-nm undoped Si layer, were fabricated at a rate of 0.02 ML/s. For all samples the Ge coverage is about 6 ML. The Ge QDs formation was controlled by reflection high energy electron diffraction when the pattern changed from streaky to spotty. Immediately after the deposition of Ge, the temperature was lowered to *T*_s _= 350-400°C and the Ge islands are covered by a 1-nm Si layer. This procedure is necessary to minimize Ge-Si intermixing and to preserve island shape and size from the effect of a further higher temperature deposition. The average Ge content of 80% in the nanoclusters was determined from Raman measurements. The samples were completed by capping a 300-nm-thick *p*^+^-doped Si layer (B, 3 × 10^18 ^cm^-3^) to form a *p*-*i*-*n *junction. The resulting devices were isolated from each other by etching 1.1-*μ*m-deep mesas.

The Si intrinsic region is not intentionally doped, nevertheless we estimated a residual MBE background doping at about 10^16 ^cm^-3 ^of *p*-type. From scanning tunneling microscopy experiments, we observe the Ge nanoislands to be approximately 15 nm in lateral size and about 1.5 nm in height. They have the form of hut clusters bounded by {105} facets. The density of the dots is about 10^11 ^cm^-2^. A typical PL spectrum of the dot sample (not shown here) is dominated by a broadband emission peaked around 820 meV.

The ER measurements were performed at room temperature using a step-scan VERTEX-70 Fourier-transform infrared spectrometer. The incident light from a tungsten halogen lamp was unpolarized and the devices were under normal incidence. A 2-kHz sine-wave voltage with a peak-to-peak amplitude of 200 mV served as an ac modulation source. Various values of reverse dc bias voltage *U*_b _was employed. The modulation reflectance of the samples was filtered with a lock-in amplifier before the Fourier transform. The phase correction recorded for background spectrum without the sample was used.

## Results and discussion

**Figure 2:**
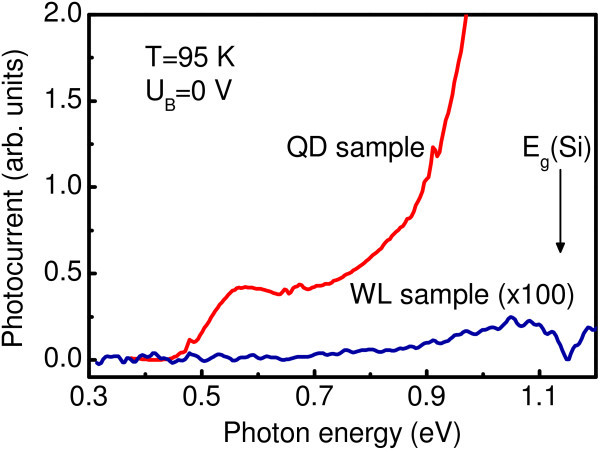
**PC spectra of the QD and WL structures measured in a short circuit configuration at *T *= 95 K**.

First we checked the photocurrent (PC) response from the devices under investigation. The applied bias was 0 V and the PC was measured in a short circuit configuration. A 2-mm-thick Si wafer serving as a filter was introduced to remove the strong PC signal due to the band-to-band transitions in the Si epitaxial layers for energies larger than 1.1 eV. Below the Si band gap energy, there is only a weak PC signal for the sample with Ge WLs (Figure 2). The spectral response of the device with QDs clearly covers a broader spectral range extending down to a half of eV.

**Figure 3:**
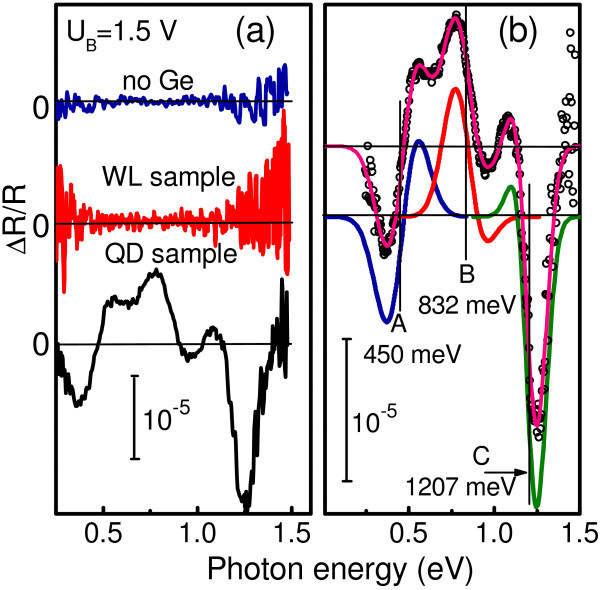
**ER spectra for different samples under investigation**. (a) ER spectra for the reference sample (no Ge was deposited) as well as for the WL and QD samples measured at reverse bias *U*_b _= 1.5 V. (b) Experimental ER spectrum (circles) and curve fit (solid lines) for the QD sample. The obtained values of the energies are represented by bars in the figure.

**Figure 4:**
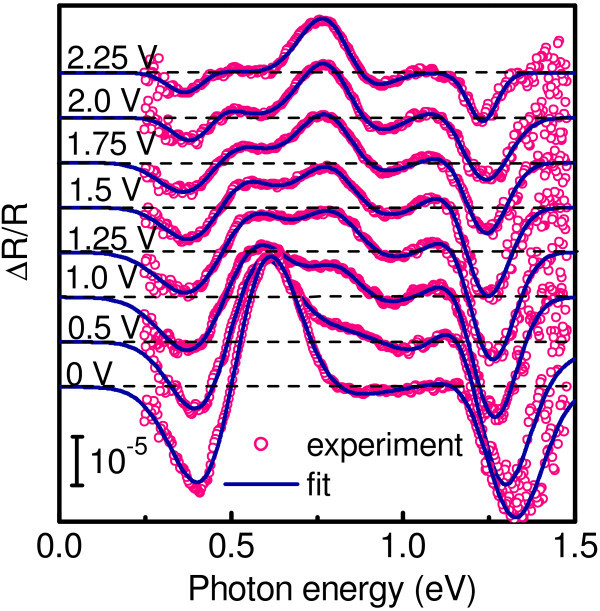
**ER spectra for QD sample under various bias voltages shifted vertically for clarity**.

Figure 3a shows typical results of ER spectroscopy. We did not observe any ER signal in a reference sample and in a sample with Ge WLs. Instead, there is an apparent, well-defined ER response from a sample containing Ge QDs. We thus associate this electromodulation with Ge nanoislands. In order to accurately determine the transition energies, we fit the data with a first-derivative Gaussian-type function [8]. The Gaussian line-shape analysis is appropriate for the inhomogeneous broadening related to the size fluctuations in ensemble of QDs. A typical curve fit is demonstrated in Figure 3b for the reverse bias of 1.5 V. As shown by the full line, a satisfactory fitting is achieved when one assumes the presence of three transitions (A, B, and C) with the energies denoted by thin vertical lines. Note that the position of the low-energy feature is close to the long-wave PC onset. Figure 4 shows the ER as a function of reverse bias applied to the diode. The low-energy reflectance modulation at 400 meV disappears at large applied voltage when the residual holes are evacuated from the dots and the Ge islands become completely depleted. We assume that this resonance corresponds to the hole intraband transition between the ground state in Ge QDs and the valence band continuum. This assignment is further supported by theoretical consideration presented below.

We consider a realistic situation when both Si matrix and Ge nanoclusters are inhomogeneously strained due to the lattice mismatch between Si and Ge. The QD is assumed to have a pyramid shape with the base oriented along the [100] and [010] directions. The pyramid base is 15 nm and the height is 1.5 nm. The pyramid lies on a 4 ML Ge WL. First, the finite element calculations of three-dimensional spatial distribution of strain components are performed. The strain modifies the band structure through the deformation potentials. A further numerical analysis of the band structure is based on a six-band **k·p **approach for the valence band and a single-band effective-mass approximation for the conduction band (CB) [9]. Coulomb interaction between the electron and hole is included into the problem.

**Figure 5:**
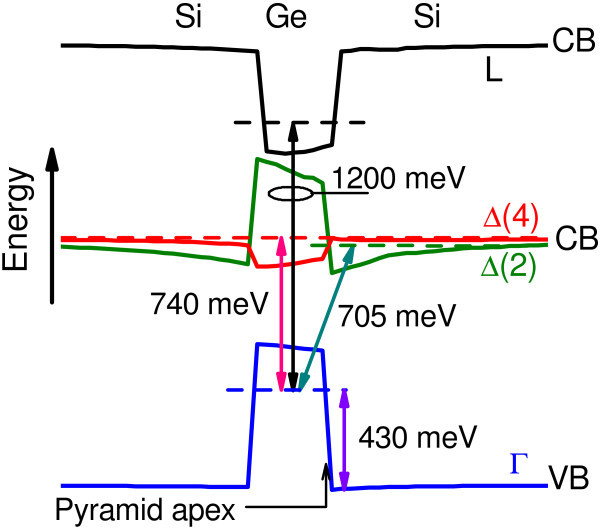
**Calculated band-edge diagram of the strained Ge pyramid in Si(001) along the growth axis with the relevant interband transitions**. For CB Δ and *L *points are shown. The electron and hole energy levels are indicated by horizontal dashed lines.

The band diagram for the three lowest interband transitions is shown in Figure 5. Due to the tensile strain, the sixfold-degenerate conduction-band minimum at the Δ point of Si around the Ge dot splits into the fourfold-degenerate in-plane Δ(4) valleys and the twofold-degenerate Δ(2) valleys along the [001] growth direction. The lowest CB edge just above and below the Ge island is formed by the Δ(2) valleys [10]. In the valence band, there is a large offset and the holes are confined inside the Ge dot, yielding type-II band-edge alignment. Since the electron and hole are spatially separated the electron-hole overlap (*f *) is as small as 0.18. The oscillator strength is greatly increased for the spatially direct interband transitions. The lowest direct transition inside the dot involves the Δ(4) CB (*f *= 0.56) and the second one includes the *L *valley (*f *= 0.86). The calculated energy difference between the spatially direct Δ(4) - Γ and spatially indirect Δ(2) - Γ transitions, 35 meV, is consistent with that obtained previously from PL spectroscopy, 34-52 meV [4]. It is worth to note that although the energies of these transitions are close to each other (0.7 eV), the oscillator strength (*∝ f *^2^) of the direct transition is larger by one order of magnitude to dominate in the absorption spectra. Therefore, it is unlikely to observe the indirect transition in absorption or reflectance experiments. However, it can be easily detected in PL measurements as they can probe selectively the ground-state emission energy.

From the comparison between the calculated transition energies (Figure 5) and the experimental ER spectrum (Figure 3) we may conclude that the low-energy resonance corresponds to a band-to-continuum hole transition in the Ge valence band. The other two modulation signals are attributed to the spatially direct transitions between the electrons confined in the *L *and Δ(4) valleys of the Ge CB, and the localized hole states at the Γ point.

**Figure 6:**
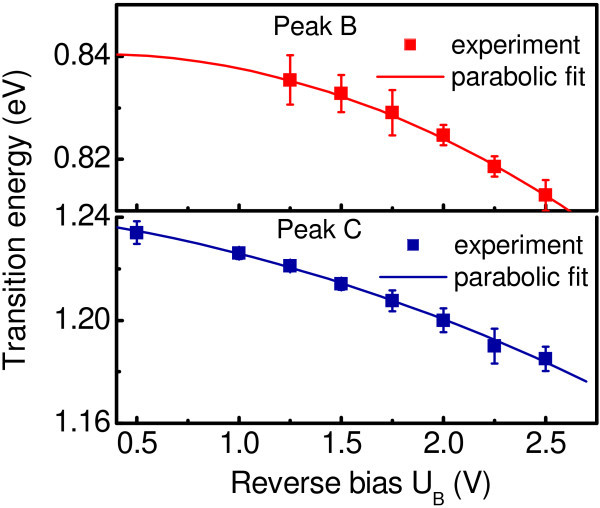
**The bias voltage dependence of the interband transition energy for peaks B and C**. The solid curves are a fit to parabolic law.

The assignment of the high-energy electromodulation signals to the direct transitions is supported by analysis of the transition energies as a function of electric field. It is known that electric field applied perpendicular to quantum wells causes the shift of the electronic transition energy, the quantum-confined Stark effect (QCSE) [11]. Type-I systems, wherein the narrow-gap dot material presents a potential well for both electron and hole, exhibit a quadratic red-shift of the transition energy [7,11,12], while there should be a linear blue-shift of the spatially indirect transition for the systems with type-II band alignment [13,14]. In Figure 6, we plot the transition energies of peaks B and C as a function of applied reverse bias. As the bias increases, both peaks are red shifted by the QCSE, implying a type-I band-edge lineup.

According to the studies by Larsson et al. [3,4] and by Adnane et al. [15], it is possible to observe the spatially direct recombination processes in the Ge/Si dot systems by using the PL measurements in specific experimental conditions which are elevated temperatures, higher excitation power [3,4] or employment of PL excitation spectroscopy [15]. Unfortunately, all these conditions are inaccessible in our experimental setup, so we did not observe direct transitions mentioned above in our PL experiments.

## Abbreviations

CB: conduction band; ER: electroreflectance; MBE: molecular beam epitaxy; PL: photo-luminescence; QDs: quantum dots; WLs: wetting layers.

## Competing interests

The authors declare that they have no competing interests.

## Authors' contributions

AY designed the study, carried out the ER measurements, participated in the simulations and coordination, and drafted the manuscript. AN prepared the samples using MBE technique. AB performed numerical analysis of the electronic structure and assisted in ER experiments. AD supervised the project work. All authors read and approved the final manuscript.
